# Development of a screening tool to predict malnutrition among children under two years old in Zambia

**DOI:** 10.1080/16549716.2017.1339981

**Published:** 2017-07-21

**Authors:** Junko Hasegawa, Yoichi M Ito, Taro Yamauchi

**Affiliations:** ^a^ School of Rehabilitation Sciences, Health Sciences University of Hokkaido, Tobetsu, Japan; ^b^ Graduate School of Medicine, Hokkaido University, Sapporo, Japan; ^c^ Faculty of Health Sciences, Hokkaido University, Sapporo, Japan

**Keywords:** Screening tool, malnutrition, children under two, developing countries, Zambia

## Abstract

**Background**: Maternal and child undernutrition is an important issue, particularly in low- and middle-income countries. Children at high risk of malnutrition should be prioritized to receive necessary interventions to minimize such risk. Several risk factors have been proposed; however, until now, there has been no appropriate evaluation method to identify these children. In sub-Saharan Africa, children commonly receive regular check-ups from community health workers. A simple and easy nutrition assessment method is therefore needed for use by semi-professional health workers.

**Objectives**: The aim of this study was to develop and test a practical screening tool for community use in predicting growth stunting in children under two years in rural Zambia.

**Methods**: Field research was conducted from July to August 2014 in Southern Province, Zambia. Two hundred and sixty-four mother-child pairs participated in the study. Anthropometric measurements were performed on all children and mothers, and all mothers were interviewed. Risk factors for the screening test were estimated by using least absolute shrinkage and selection operator analysis. After re-evaluating all participants using the new screening tool, a receiver operating characteristic curve was drawn to set the cut-off value. Sensitivity and specificity were also calculated.

**Results**: The screening tool included age, weight-for-age Z-score status, birth weight, feeding status, history of sibling death, multiple birth, and maternal education level. The total score ranged from 0 to 22, and the cut-off value was eight. Sensitivity and specificity were 0.963 and 0.697 respectively.

**Conclusions**: A screening tool was developed to predict children at high risk of malnutrition living in Zambia. Further longitudinal studies are needed to confirm the test’s validity in detecting future stunting and to investigate the effectiveness of malnutrition treatment.

## Background

Malnutrition is defined as a lack of sufficient nutrition to meet growth demands, body maintenance, and activity. Maternal and child undernutrition is the underlying cause of childhood malnutrition, which accounts for nearly one-third of deaths among children younger than five years of age [1]. The first few years of life are particularly important because vital development occurs in all domains. Particularly with regard to brain development, even small interruptions in the ontogenetic process can have long-term effects on the brain’s structural and functional capacity [2]. Major damage caused by malnutrition is irreversible and causes reduced intelligence and physical capacity, which in turn reduce productivity, thereby slowing economic growth [3]. Growth failure prior to 12 months of age affects adult stature, which supports the importance of optimal nutritional status in infancy [4]. Currently, a global movement called *Scaling Up Nutrition* has been established to unite researchers with members of government, civil society, international non-governmental organizations, and businesses in collective efforts to improve nutrition. The first 1000 days of life, called the ‘window of opportunity’ – from fertilization up to the second birthday – are widely regarded as an important period within which to prevent stunting [5–7].

Malnourishment in the early stages of infancy can easily develop into moderate or severe malnutrition, particularly in developing countries. To prevent child malnutrition, several nutritional screening tools for predicting outcomes for hospitalized children are available [8–11]. However, to our knowledge, few such tools target children in the community setting. Among children living in the community, stunting is a major concern.

Several indices derived from anthropometric measurements can be used to evaluate nutritional imbalance resulting in undernutrition. Children whose height-for-age Z-score is two standard deviations below mean are considered ‘stunted’, which indicates linear growth retardation and cumulative growth deficits [12]. Various factors have been associated with child nutritional status, with several studies demonstrating an association between maternal height and child nutritional status [13–15]. It has been previously hypothesized that growth-stunted women may provide a nutrient-restricted uterine environment, leading to an inadequate supply of nourishment for the baby’s growth, resulting in infant stunting [16]. Arguments have also been made in favour of exclusive breastfeeding, with a shorter period of exclusive breastfeeding leading to a greater likelihood of stunting [17]. The World Health Organization (WHO) recommends exclusive breastfeeding until six months of age, which is widely promoted in Zambia via antenatal clinics and other health-promotion activities. The effect of maternal education on child stunting has also been investigated, with Abuya reporting that maternal education was negatively correlated with child stunting [18].

Community-dwelling children in sub-Saharan Africa, as well as those in hospitals, are at risk of malnutrition; therefore, they should be screened and receive interventions as necessary. However, health facilities in those countries face limitations, such as lack of measurement equipment and human resources, including community health workers (CHW), who play an important role in developing countries [19]. Nonetheless, semi-professionals working in these facilities effectively contribute to the health of the communities, including child health. However, the quality of their services is not always assured. Continuous supervision is necessary, and there is a need for assessment tools that are simple and easy to administer. However, to our knowledge, such assessment methods are lacking.

Regular health check-ups represent an opportunity for screening children at high risk of malnutrition, but there are generally insufficient qualified healthcare personnel in sub-Saharan Africa. Development of a simple and easy assessment tool that meets satisfactory standards would help to overcome this challenge. The aim of this study was to develop and test a practical screening tool for community use in predicting growth stunting in children under two years in rural Zambia.

## Methods

### Study area, study period, and sampling

A cross-sectional study was conducted from July to August 2014 at Sinazeze Rural Health Centre (SRHC), located in Sinazeze Township in Sinazongwe district, Southern Province, Zambia. Most rural health centres in Zambia have remote bases called outreach clinics to provide individuals living in rural areas with medical services. SRHC has 12 outreach clinics providing care to 12,000 people [20].

A field study was conducted using convenience sampling. Participants were children aged 0–24 months and their mothers who visited SRHC or outreach clinics for vaccinations or regular check-ups. Two hundred and sixty-six mother-child pairs participated in the study. Two mother-child pairs were excluded, including a mother with language difficulties and a child suspected of having Down’s syndrome. Ultimately, 264 mother-child pairs were included in the study.

### Measurement and variables

#### Anthropometric measurements

Children’s length and weight were measured using an infant scale with a length meter (Seca Model 336, 232, Germany) [21]. Mothers’ height and weight were measured using a mobile stadiometer (Seca Model 213, Germany) and digital weight measure (Tanita HD-654, Japan), respectively.

#### Interviews

**Figure 1.**
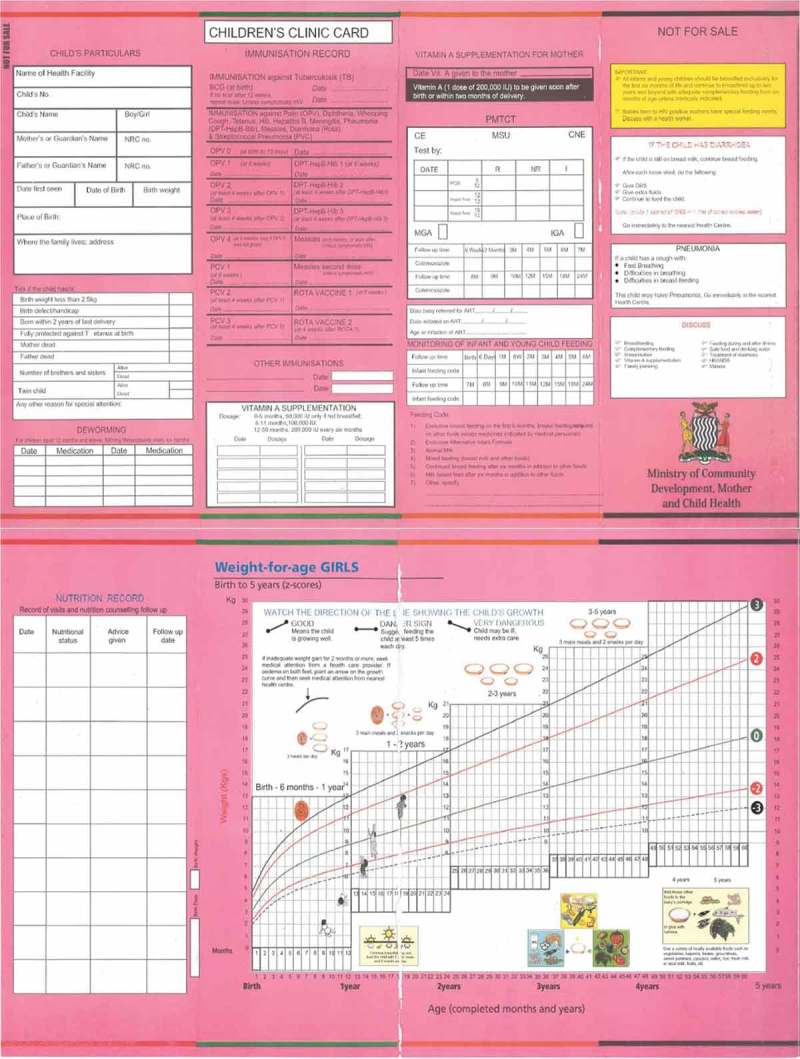
Maternity health record book (for girls).

We conducted semi-structured interviews with all mothers who participated in this study to assess three domains: child’s health history, mother’s health and knowledge, and household living environment. The interviews were conducted in English or Tonga, a language commonly spoken in the study area. The interviewer, an individual fluent in both English and Tonga, was provided with training in advance. We also referred to the maternity health record book (Figure 1), which is distributed to the family of each child to document their health from birth until preschool. The book includes columns to list the child’s personal information, such as birth date, sex, name, birth weight, and delivery status. This book is not always used appropriately; for example, some children born at home do not receive a record book in a timely manner and some contain omissions, but when this record book was available, we referred to it in order to establish the accuracy of the interview.

### Variables

To allow for ease of use in Zambian rural locations, only variables that were easily assessed without specialized skills were selected. Continuous variables were reclassified as categorical variables for convenience of use in the field. For example, weight-for-age Z-score (WAZ) was considered dichotomously as either less than zero or as zero and higher. Weight measurements are normally plotted on a chart in each individual’s maternity health record (Figure 1), with a growth curve included, so as to identify whether the WAZ was less than or at least zero. Current feeding status was divided into the recommended or non-recommended method, with reference to WHO recommendations [22]. Non-recommended feeding status included children less than six months of age who had already started solid food or children aged 6–24 months who had not yet started complementary food or who had already been weaned from breastmilk.

The following variables were used to derive the screening tool: sex, age (<12 or 12–24 months), underweight (WAZ <0 or ≥0), birth weight (≥2500 g or <2500 g, when unavailable considered to be <2500 g), place of delivery (health facility or elsewhere), number of siblings, number of visits to an antenatal clinic (never or at least once), current feeding status (recommended method or not), mother’s marital status (in a relationship or other), history of sibling death (yes or no), head of household (mother or other), drinking water (recommended hygienic source or other), toilet facility (recommended facilities or other), mother’s educational status (primary school or less or at least some secondary school), and multiple birth (yes or no).

### Outcome

To develop the screening tool, stunting and length-for-age Z-score (LAZ) were defined as the outcomes for statistical analysis. LAZ was calculated using WHO Standards 2006 [23,24]. Stunting was defined as two standard deviations or more below the median length for age of the reference population (LAZ < −2).

### Statistical analysis

Multivariate logistic regression analysis using the least absolute shrinkage and selection operator (LASSO) selection method was performed to estimate significant risk factors for predicting malnutrition [25]. Leave-one-out cross-validation was used as a validation method. To develop a screening tool to predict malnutrition based on the outcomes of LAZ and stunting, we assigned each category of risk factor with one of the following point scores in accordance with the method of Lindström and Tuomilehto [26]: at β-coefficient = 0.01–0.2, the score was one; at β = 0.21–0.8, the score was two; at β = 0.81–1.2, the score was three; at β = 1.21–2.2 the score was four; and at β > 2.2, the score was five. The reference category of each variable was given a score of zero. We developed three screening tools: two based on the results of stunting and LAZ, the stunting model and LAZ model, respectively, and the other LAZ and stunting mixed model (mixed model). The mixed model consisted of the higher value of the β-coefficients of both the LAZ and stunting, since these two models differed slightly. The total score of the screening tool was calculated as the sum of the individual scores. The maximum score was 14 for the stunting model, 19 for the LAZ model, and 22 for the mixed model. A receiver operating characteristic (ROC) curve was drawn for the screening tool to assess predictive performance with the calculation of the area under the curve (AUC).

## Results

The characteristics of study participants are summarized in Table 1. Approximately 10% of children who participated in the study were identified as stunted. The prevalence of stunted children under five years of age reported in the Zambia Demographic and Health Survey 2013–14 [12] was 27%, considerably higher than the current finding. Regarding current feeding status, the majority of mothers were breastfeeding, which reflects WHO recommendations for children aged 0–24 months. Among all participating children, 50.8% were less than six months old and therefore it was recommended that they be exclusively breastfed. More than 90% of mothers with a child under six months old exclusively breastfed, while less than 10% of children less than six months old had started solid food or another non-milk liquid. Similarly, among children 6–24 months old, for whom it is recommended to start complementary food and continue breastfeeding, almost 10% had not yet started complementary food or had already been weaned from breast milk. Thus, nearly 10% of participants were considered as not following WHO recommendations. Eighteen percent of children had an older sibling who had died. More than 80% of mothers had at least primary-level education, and 16.4% had received at least some secondary-level education.

**Table 1.** Characteristics of the study participants (*N* = 264).

Factor	Category	Number of participants	(%)
Child’s nutritional status	LAZ < −2 (stunted)	27	(10.2)
	LAZ ≥ −2	237	(89.8)
	WAZ < −2 (underweight)	20	(7.6)
	WAZ ≥ −2	244	(92.4)
	WLZ < −2 (wasting)	9	(3.4)
	WLZ ≥ −2	255	(96.6)
Child age (months)	<6	134	(50.8)
	6–8	35	(13.3)
	9–11	40	(15.2)
	12–17	32	(12.1)
	18–24	23	(8.7)
Child’s sex	Female	127	(48.1)
	Male	137	(51.9)
Birth weight (g)	≥2500	172	(65.2)
	<2500	25	(9.5)
	NA	67	(25.4)
Current feeding	Breastmilk only	126	(47.7)
	Breastmilk and solid food	128	(48.5)
	Solid food only	10	(3.8)
Place of delivery	Health facility	249	(94.3)
	Home or other place	15	(5.7)
Mother’s age	15–19	51	(19.3)
	20–29	126	(47.7)
	30–39	77	(29.2)
	40–49	10	(3.8)
Mother’s BMI	<18.5	7	(2.7)
	18–25.0	202	(76.5)
	≥ 25	55	(20.8)
Pregnancy intention	Wanted now	249	(94.3)
	Never wanted	15	(5.7)
Antenatal visits	Never	8	(3)
	At least once	252	(95.5)
Mother’s educational status	Primary or less	221	(83.7)
	Secondary +	43	(16.3)
Father’s education level	Primary or less	174	(65.9)
	Secondary +	68	(25.8)
	NA	22	(8.3)
History of sibling’s death	Death (−)	216	(81.8)
	Death (+)	48	(18.2)
Source of drinking water	Piped	75	(28.4)
	Other improved	148	(56.1)
	Non-improved	41	(15.5)
Type of toilet	Improved	117	(44.3)
	Non- improved	147	(55.7)
Headship of the household	Father	238	(90.2)
	Mother	7	(2.7)
	Others	19	(7.2)

**Figure 2.**
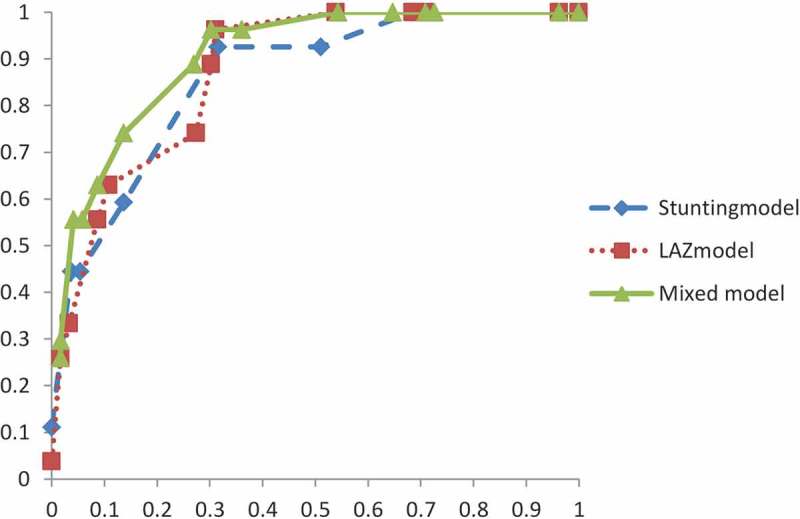
Receiver operating characteristic curves of each screening tool.

Significant predictors of stunting and LAZ, according to LASSO analysis, are listed in Table 2. There was a high degree of agreement between stunting and LAZ in the detection of significant predictors. Predictors common to both outcomes were age, WAZ <0, low birth weight or missing data, and non-recommended feeding status. Multiple birth, history of sibling death, and mother’s education level were significant predictors only of LAZ. The β-coefficient was mostly higher among the stunting results. All risk factors in the three different screening tools and points derived for each category are shown in Table 3. Age, low birth weight, and current feeding status showed different scores. The total score ranged from 0 to 14 in the stunting model, from 0 to 19 in the LAZ model, and from 0 to 22 in the mixed model. A greater number of points indicated a higher risk of malnutrition. ROCs of each screening tool are shown in Figure 2. All three curves had satisfactory AUC values. Based on the ROC curve, the mixed model was the most suitable among the three models (AUC, 0.904). A score of eight in the mixed model was determined as the threshold at which the Youden index reached its peak. At the determined threshold, the sensitivity, specificity, positive predictive value, and negative predictive value were 0.963, 0.697, 0.263, and 0.994, respectively.

**Table 2.** Significant predictor of LAZ and stunting.

	LAZ		Stunting	
Predictor	β-coefficient	*p* value	β-coefficient	*p* value
Age (1year or more)	0.88	<0.001	2.10	<0.001
WAZ <0	1.28	<0.001	2.04	<0.001
Low birth weight or data missing	0.49	<0.001	1.12	0.02
Unrecommended feeding status	0.37	0.05	1.11	0.04
Multiple birth	1.54	<0.001	1.87	0.07
History of sibling’s death	0.29	0.05	0.32	0.61
Mother’s education level (secondary school or higher)	0.29	0.04	1.03	0.16

**Table 3.** Points assigned to predict stunting.

	Stunting		LAZ		Mixed	
Risk factors	β-coefficient	Point	β-coefficient	Point	β-coefficient	Point
**Age**						
<1 year		0		0		0
≥1 year	2.10	4	0.88	3	2.10	4
**WAZ**						
<0	2.04	4	1.28	4	2.04	4
≥0		0		0		0
**Low birth weight**						
Yes	1.12	3	0.49	2	1.12	3
No		0		0		0
**Current feeding status**						
Recommended		0		0		0
Unrecommended	1.11	3	0.37	2	1.11	3
**Multiple birth**						
Yes			1.54	4	1.54	4
No				0		0
**History of sibling’s death**						
Yes			0.29	2	0.29	2
No				0		0
**Mother’s education status**						
Primary-			0.29	2	0.29	2
Secondary+				0		0
**Total score**		14		19		22

Abbreviations: LAZ, length-for-age Z-score; WAZ, weight-for-age Z-score.

## Discussion

The screening tool was developed for use in Zambia to predict stunting among children under two in a community setting. The screening tool, employing a mixed model, demonstrated satisfactory predictive ability for the incidence of stunting. The screening tool consists of seven items: age, WAZ, birth weight, current feeding status, multiple birth, history of sibling death, and maternal education. This study focused on Zambia as a target area. However, a tool that reflects different areas could be developed using the same method. Choosing easily assessable variables for the analysis, we developed a tool appropriate for use in a Zambian rural location where semi-professional health workers have an active role in child health.

Some variables included in the tool appear unrelated to nutritional status. For example, low maternal educational level was associated with a higher level of child stunting. Previous studies have shown low maternal educational status (primary school or less) to be related to decreased LAZ [18,27]. The effect of low parental education on a child’s nutritional status can be attributed to a lack of health-related knowledge and, thus, reduced health-seeking behaviour. For example, it is reported that in Morocco, schooling does not contribute to a mother’s health knowledge directly, but that literacy and numeracy skills acquired in school were useful, suggesting that education plays an important role in raising healthy children [28].

Another variable included in the tool was current feeding status, which includes exclusive breastfeeding and complementary food. The manner in which points for current feeding status was scored is consistent with WHO recommendations [22]. The finding that feeding status was predictive of stunting indicates that breast milk is an important energy source, even after complementary feeding has started. This is probably because breast milk serves as a critical source of energy and nutrients during illness and contains protective factors against disease. Even if a mother is undernourished, this has been shown to have little effect on the volume or composition of breast milk, unless malnutrition is severe [1,22]. Accordingly, when complementary food cannot be secured, breast milk plays an important role in child nutrition. Residents in the study area rely on maize, which is a complementary food for infants. Cereal grains must be considered not only as a source of energy but also as a source of protein. However, the protein quality of maize is limited by deficiencies in some essential amino acids [29]. Breast milk is recommended in addition to maize as it adds nutritive value; thus, weaning from breast milk too early may adversely affect child nutrition status.

At the cut-off point of ≥8, the present screening test has higher sensitivity and lower specificity than previously reported tools for hospitalized children: 70% sensitivity and 91% specificity for one such tool [8] and 59% sensitivity and 92% specificity for another [30]. Given the serious negative effects of malnutrition among children under two, sensitivity is more important than specificity.

### Strengths and limitations

The screening tool developed in the current study requires verification for its results to be more generalizable given the restricted target area. Furthermore, some potentially important confounding factors, such as household economic status, were not considered in this study. However, there are three distinguishing features. Firstly, it targets community-dwelling children and considers their past health and family environment. Secondly, the test is simple and easy to administer, which allows non-professional CHW to use it without adding further burden to their work. Thirdly, it demonstrated high sensitivity and specificity.

## Conclusion

Child malnutrition is a major health problem in Zambia. To prevent malnutrition among children under two, identifying risk of growth stunting is a crucial first step. Because CHW play an important role in child growth monitoring, we developed a malnutrition screening tool that (1) helps identify high-risk children at early stage, (2) requires information that is relatively easy to access and assemble in community settings, and (3) allows for ease of judgement and decision-making.

Further research, for example in the form of a longitudinal studies which target several communities, is needed to confirm the test’s validity in detecting future stunting. The efficacy of early identification of high-risk status for early intervention and prevention of growth stunting should be verified.

## Paper context

Stunting among young children negatively influences their future health. Detection and intervention during the first 1000 days is most critical. To reduce child stunting, the efficacy of multi-sectoral interventions involving community health workers should be investigated. We developed and tested a tool for the early detection of potentially malnourished children living in rural community settings in Zambia. This tool is designed for easy use by community health workers in rural settings in developing countries.

## References

[1] Black RE, Allen LH, Bhutta ZA, et al Maternal and child undernutrition: global and regional exposures and health consequences. Lancet. 2008;371:243–8. 18207566 10.1016/S0140-6736(07)61690-0

[2] Grantham-McGregor S, Cheung YB, Cueto S, et al Child development in developing countries 1. Developmental potential in the first 5 years for children in developing countries. Lancet. 2007;369:60–70. 17208643 10.1016/S0140-6736(07)60032-4 PMC2270351

[3] The World Bank 2006 Repositioning nutrition as central to development: a strategy for large scale action. Washington (DC): World Bank [cited 2015 6 17]. Available from: http://www.unhcr.org/45f6c4432.pdf

[4] Stein AD, Wang M, Martorell R, et al Growth patterns in early childhood and final attained stature: data from five birth cohorts from low-and middle-income countries. Am J Hum Biol. 2010;22:353–359. 19856426 10.1002/ajhb.20998 PMC3494846

[5] Bezanson K, Isenman P. Policy Brief: scaling up nutrition: a framework for action. Food Nutr Bull. 2010;31:178–186. 20461915 10.1177/156482651003100118

[6] Bryce J, Coitinho D, Darnton-Hill I, et al Maternal and child undernutrition: effective action at national level. Lancet. 2008;371:510–526. 18206224 10.1016/S0140-6736(07)61694-8

[7] National Food and Nutrition Commission of Zambia 2015 National food and nutrition strategic plan for Zambia 2011-2015: with a multi-sector strategic direction on First 1000 Most Critical Days to prevent child stunting. Lusaka: National Food and Nutrition Commission of Zambia [cited 2015 6 13]. Available from: http://www.scalingupnutrition.org/wp-content/uploads/2013/02/Zambia_NFNC-Stratergic-Plan-2011-2015.pdf

[8] McCarthy H, Dixon M, Crabtree I, et al The development and evaluation of the Screening Tool for the Assessment of Malnutrition in Paediatrics (STAMP (C)) for use by healthcare staff. J Hum Nutr Diet. 2012;25:311–318. 22568534 10.1111/j.1365-277X.2012.01234.x

[9] Hulst JM, Zwart H, Hop WC, et al Dutch national survey to test the STRONGkids nutritional risk screening tool in hospitalized children. Clin Nutr. 2010;29:106–111. 19682776 10.1016/j.clnu.2009.07.006

[10] Gerasimidis K, Keane O, Macleod I, et al A four-stage evaluation of the Paediatric Yorkhill Malnutrition Score in a tertiary paediatric hospital and a district general hospital. Br J Nutr. 2010;104:751–756. 20398432 10.1017/S0007114510001121

[11] Sermet-Gaudelus I, Poisson-Salomon AS, Colomb V, et al Simple pediatric nutritional risk score to identify children at risk of malnutrition. Am J Clin Nutr. 2000;72:64–70. 10871562 10.1093/ajcn/72.1.64

[12] Central Statistical Office Zambia Zambia Demographic and Health Survey 2013-14. Lusaka; 2014 [cited 2015 5 28]. Available from: https://www.dhsprogram.com/pubs/pdf/FR304/FR304.pdf

[13] Ozaltin E, Hill K, Subramanian SV Association of maternal stature with offspring mortality, underweight, and stunting in low- to middle-income countries. JAMA. 2010;303:1507–1516. 20407060 10.1001/jama.2010.450 PMC3100588

[14] Addo OY, Stein AD, Fall CH, et al Maternal height and child growth patterns. J Pediatr. 2013;163:549–554. 23477997 10.1016/j.jpeds.2013.02.002 PMC3711792

[15] Subramanian SV, Ackerson LK, Davey Smith G, et al Association of maternal height with child mortality, anthropometric failure, and anemia in India. JAMA. 2009;301:1691–1701. 19383960 10.1001/jama.2009.548 PMC3095774

[16] Santos Felisbino-Mendes M, Villamor E, Velasquez-Melendez G Association of maternal and child nutritional status in Brazil: a population based cross-sectional study. PLoS One. 2014;9:e87486. 10.1371/journal.pone.0087486 PMC3901750 24475297

[17] Fikadu T, Assegid S, Dube L Factors associated with stunting among children of age 24 to 59 months in Meskan district, Gurage Zone, South Ethiopia: a case-control study. BMC Public Health. 2014;14:800–807. 25098836 10.1186/1471-2458-14-800 PMC4131046

[18] Abuya BA, Ciera J, Kimani-Murage E Effect of mother’s education on child’s nutritional status in the slums of Nairobi. BMC Pediatr. 2012;12:80. 22721431 10.1186/1471-2431-12-80 PMC3444953

[19] Lehmann U, Sanders D Community health workers: what do we know about them? The state of the evidence on programmes, activities, costs and impact on health outcomes of using community health workers. Geneva: World Health Organization; 2007 [cited 2016 9 10]. Available from: http://www.who.int/hrh/documents/community_health_workers.pdf

[20] Zambian Ministry of Health The 2012 list of health facilities in Zambia: preliminary Report, v15. Lusaka: Ministry of Health; 2013 Available from: http://www.moh.gov.zm/docs/facilities.pdf

[21] Weiner JS, Lourie JA Practical human biology. London: Academic Press Inc; 1981.

[22] World Health Organization Infant and young child feeding. Geneva; 2011 [cited 2015 5 30]. Available from: http://whqlibdoc.who.int/publications/2009/9789241597494_eng.pdf

[23] World Health Organization WHO child growth standards: length/height-for-age, weight-for-age, weight-for-length, weight-for-height and body mass index-for age. Geneva: World Health Organization; 2006.

[24] WHO Working Group Use and interpretation of anthropometric indicators of nutritional status. Bull World Health Organ. 1986;64:929–941. 3493862 PMC2490974

[25] Tibshirani R Regression selection and shrinkage via the lasso. J R Stat Soc Series B Stat Methodol. 1996;58:267–288.

[26] Lindström J, Tuomilehto J The diabetes risk score: a practical tool to predict type 2 diabetes risk. Diabetes Care. 2003;26:725–731. 12610029 10.2337/diacare.26.3.725

[27] El Taguri A, Betilmal I, Mahmud SM, et al Risk factors for stunting among under-fives in Libya. Public Health Nutr. 2009;12:1141–1149. 18789172 10.1017/S1368980008003716

[28] Glewwe P Why does mother’s schooling raise child health in developing countries? Evidence from Morocco. J Hum Resour. 1999;34:1.

[29] Food and agriculture organization of the United Nations Maize in human nutrition. Rome: Food and Agriculture Organization; 1992. 6086142

[30] Wiskin AE, Owens DR, Cornelius VR, et al Paediatric nutrition risk scores in clinical practice: children with inflammatory bowel disease. J Hum Nutr Diet. 2012;25:319–322. 22591201 10.1111/j.1365-277X.2012.01254.x

